# Statins Mitigate Stress-Related Vascular Aging and Atherosclerosis in *apoE*-Deficient Mice Fed High Fat-Diet: The Role of Glucagon-Like Peptide-1/Adiponectin Axis

**DOI:** 10.3389/fcell.2021.687868

**Published:** 2021-07-21

**Authors:** Yanna Lei, Qingsong Cui, Guang Yang, Limei Piao, Aiko Inoue, Hongxian Wu, Xiang Li, Masafumi Kuzuya, Xian Wu Cheng

**Affiliations:** ^1^Department of Intensive Care Unit, Yanbian University Hospital, Yanjin, China; ^2^Department of Cardiology and Hypertension, Yanbian University Hospital, Yanjin, China; ^3^Institute of Innovation for Future Society, Nagoya University Graduate School of Medicine, Nagoya, Japan; ^4^Department of Cardiology, Shanghai Institute of Cardiovascular Disease, Zhongshan Hospital, Fudan University, Shanghai, China; ^5^Department of Community Healthcare & Geriatrics, Nagoya University Graduate School of Medicine, Nagoya, Japan

**Keywords:** chronic stress, statins, atherosclerosis, inflammation, adiponectin

## Abstract

**Objectives:**

Exposure to chronic psychosocial stress is a risk factor for atherosclerotic cardiovascular diseases. Given that the 3-hydroxy-3-methylglutaryl-coenzyme reductase inhibitor statins prevent atherogenesis, we evaluated whether pitavastatin prevents chronic stress- and high fat diet-induced vascular senescence and atherogenesis in apolipoprotein *E*-deficient (*ApoE*^–/–^) mice, with a special focus on glucagon-like peptide-1 (GLP-1)/adiponectin (APN) axis.

**Methods and Results:**

6-week-old *ApoE*^–/–^ mice loaded a high-fat diet were randomly assigned into non-stress (*n* = 12) and stress (*n* = 13) groups for 12 weeks. Non-stress control mice were left undisturbed. Chronic stress accelerated high fat diet-induce arterial senescence and atherosclerotic plaque growth. The chronic stress lowered the levels of circulating GLP-1 as well as adipose and plasma APN. As compared with the stress alone mice, the pitavastatin-treated mice had reduced macrophage infiltration, elastin fragments, and increased plaque collagen volume, and lowered levels of osteopontin, toll-like receptor-2/-4, macrophage chemoattractant protein-1, C-X-C chemokine receptor-4, p47^*phox*^, p47^*phox*^, gp91^*phox*^, cathepsins S, p16, and p21, mRNAs and/or proteins. Pitavastatin increased plasma GLP-1 and APN levels and suppressed matrix metalloproteinase-2/-9 gene expressions and activities in the aortas. Finally, the protective effect of pitavastatin was abrogated by APN blocking.

**Conclusion:**

These findings suggested that the pitavastatin-mediated pleiotropic vasculoprotective effects are likely attributable, at least in part, to the elevation of GLP-1 and APN levels and the inhibition of diet-induced plaque inflammation, oxidative stress, and proteolysis in *ApoE*^–/–^ mice received chronic stress conditions.

## Introduction

Accumulating evidence indicated that chronic stress is involved in metabolic and inflammatory cardiovascular disorders (Bernberg et al., 2012; Heidt et al., 2014). The mechanisms underlying chronic stress-related diseases have thus become a research focus. At present, most of the evidence suggests that the pathogenic effect of chronic stress is exerted mainly on the hypothalamic-pituitary-adrenal axis and/or the body’s sympathetic nervous system, followed by the induction of disorders such as atherosclerosis (Barik et al., 2013; Cox et al., 2014; Heidt et al., 2014). Recently we demonstrated that chronic stress can prompt the formation of atherosclerosis by accelerating inflammation and oxidative stress (Lei et al., 2017; Yang et al., 2017). Despite the large number of studies that have been performed, the precise mechanisms are not yet clear, and effective strategies to cope with chronic stress-related diseases remain to be established.

Statins are traditional anti-atherosclerosis drugs, although they are normally used against low-density lipoprotein cholesterol (LDL-C); statins have also been shown to have an anti-atherosclerotic effect partly by inhibiting inflammation and oxidative stress (Lin et al., 2015). Statins have been widely adopted as a clinical atherosclerosis treatment, but the question of whether statins also have a beneficial effect on chronic stress-related atherosclerosis has not been resolved. We conducted the present study to explore the potential mechanisms involved in chronic stress-related atherosclerosis formation, focusing on inflammation and oxidative stress. We also investigated whether the traditional anti-atherosclerosis drug pitavastatin can ameliorate this stress-related atherosclerosis and its mechanisms, with a special focus on glucagon-like peptide-1 (GLP-1)/adiponectin (APN) axis.

## Materials and Methods

### Animals

Four-week-old male *ApoE*^–/–^ mice (21–24 g body weight; KOR/StmSlc-Apoe^*shl*^, BALB/c background) were purchased from Chubu Science and Material Science Company (SLC, Hamamatsu, Japan). For their adaptation to the new environment, the mice were all fed a standard diet for 2 weeks and housed two per cage under standard conditions (50 ± 5% humidity, 23 ± 1°C). The animal experimental protocols were approved by the Institutional Animal Care and Use Committee of Nagoya University (Protocol No. 27304) and of Yanbian University (Protocol No. 2018-10) and performed according to the Guide for the Care and Use of Laboratory Animals published by the U.S. National Institutes of Health.

### Mouse Immobilized Stress Protocol and Tissue Correction

The 6-week-old male *ApoE*^–/–^ mice (*n* = 25) were randomly assigned into the following two groups for the first phase of the experiment: The control group (HF-C, *n* = 12) received a high-fat (HF) diet (21.00% fat and 0.15% cholesterol) only, and the stress group (HF-S, *n* = 13) received the same HF diet and were subjected to restraint stress (12 weeks) (Lei et al., 2017). For the second phase of the experiment, the *ApoE*^–/–^ mice fed a HF diet were divided to another two groups: Stress 2 group (Stress): the mice subjected to restraint stress for 12 weeks as mentioned above, and the stress + pitavastatin group (S-Pis): Except for HF diet and restraint stress (12 weeks), the mice were treated with pitavastatin (1 mg/kg/d). The pitavastatin used in this experiment was provided by Kowa Pharmaceutical Co. Ltd. (Nagoya, Japan). For the APN deletion examinations, the mice (*n* = 10) fed with HF-diet were divided to one of two groups and gave pitavastatin + control rabbit IgG (Control, 450 μg/kg/d, ab27472, Cambridge, United Kingdom) or pitavastatin plus neutralizing rabbit APN antibody (S-NAPN, 450 μg/kg/d, ab3455) given by subcutaneous injection every week under chronic stress conditions for 8 weeks.

After euthanasia of the *ApoE*^–/–^ mice by means of an overdose of pentobarbital (50 mg/kg; Dainippon Pharmaceutical, Osaka, Japan) at the end of stress/no stress treatment, blood samples were collected just before perfusion into syringes containing heparin. After perfusion with 0.01 M phosphate-buffered saline (PBS, pH 7.4), the aorta tissues were collected for biological analyses and histological characterization analyses.

### Histological Analysis

The cross-sections of the aortas were examined as we described (Cheng et al., 2011). The heart was sliced in a plane between the lower tips of the left and right atria, and the upper portion of the heart was isolated. The hearts were kept in 4% formalin. Serial sections (3-μm thick at 15-μm intervals) were isolated on slides for morphological analysis and immunostaining.

### Quantitative Real-Time PCR

Total RNA was extracted from the aortic tissues and preadipocytes with the use of the RNeasy Fibrous Tissue MiNi-Kit (Qiagen, Hilden, Germany) and was subjected to reverse transcription following the manufacturer’s instructions. The cDNA was generated by the SuperScript III CellsDirect cDNA Synthesis kit (Invitrogen, Carlsbad, CA, United States). A polymerase chain reaction (PCR) was done by the ABI 7300 Real-time PCR System (Applied Biosystems, Foster City, CA, United States). The expression of glyceraldehyde 3-phosphate dehydrogenase (GAPDH) was used as an internal standard for each targeted gene levels. The primer sequences for the macrophage chemoattractant protein-1 (MCP-1), toll-like receptor-2/-4 (TLR-2/-4), p47^*phox*^, p47^*phox*^, gp91^*phox*^, cathepsins S (Cat S), matrix metalloproteinase-2/-9, (MMP-2/-9), and C-X-C chemokine receptor-4 (CXCR-4) are listed in Table 1.

**TABLE 1:** Primer sequences for mice used for quantitative real-time PCR.

**Genes name**	**Forward primers**	**Reverse primers**
MMP-2	CCCCATGAAGCCTTGTTTACC	TTGTAGGAGGTGCC CTGGAA
MMP-9	CCAGACGCTCTTCGA GAACC	GTTATAGAAGTGGC GGTTGT
Cat S	GTGGCCACTAAAGG GCCTG	ACCGCTTTTGTAGAAGAAGA AGGAG
gp 91	ACTTTCCATAAGATGGTAGC TTGG	GCATTCACACACCAC TCAACG
MCP-1	GCCCCACTCACCTGC TGCTACT	CCTGCTGCTGGTGATCC TCTTGT
TLR-2	AAGAAGCTGGCATTC CGAGGC	CGTCTGACTCCGAGGG GTTGA
TLR-4	AGTGGGTCAAGGAACA GAAGCA	CTTTACCAGCTCATTT CTCACC
CXCR- 4	CCACCCAGGACAGTGTGACTCT AA	GATGGGATTTCTGTAT GAGGATT
p47^*phox*^	CTGAGGGTGAAGCCA TTGAGG	GCCGGTGATATC CCCTTTCC
p67^*phox*^	AACTACCTGGAGCC AGTTGAG	AATTAGGAGGTGGTGGAAT ATCGG

### Immunohistochemistry

The 3-μm-thick paraffin sections of aortas were H&E stained as we described (Cheng et al., 2011). Corresponding slides were immunostained with mouse antibodies against macrophages (CD68; 1:100, Chemicon, Billerica, MA, United States), mouse antibodies against osteopontin (1:125, Sigma-Aldrich, St. Louis, MO, United States), rabbit antibodies against alpha-smooth muscle actin (α-SMA; 1:100, Neo Markers, Fremont, CA, United States). Elastin and collagen contents were quantified using the Elastica van Gieson (EVG)-stained and picrosirius red (PSR)-stained positive areas. Images of sections stained for macrophages, elastin, osteopontin, α-SMC, and collagen were quantified with ImagePro software (BZ9000 Analysis, Keyence, Japan). Six cross-sections of each aortas were calculated and averaged for each mouse. The results are expressed as the percentage of intima area that contained the lesion.

### Oil Red O Staining and Senescence-Associated β-Galactosidase (β-Gal) Staining

The whole aortas from mice of each group were selected. We first removed the extra fatty and adventitia tissue from the vessels using forceps and scissors. The vessels were then incised longitudinally along the artery and fixed in formalin overnight for oil red O staining, as described in our previous study (Lei et al., 2017). Images of each vessel were obtained, and the extent of atherosclerosis was determined with the ImagePro software. β-Gal staining and quantitative analyses were made as described with our previous study (Lei et al., 2017).

### ELISA and Biochemical Assays

According to manufacturers’ instructions, the plasma GLP-1 and adiponectin (APN) levels were evaluated using the commercially available ELISA kits (GLP-1, Cat. EZGLP1T-36K; EMD Millipore, Billerica, MA, United States; APN, Cat. MRP300; R&D Systems, Minneapolis, MN, United States). The levels of the mouse plasma non-esterified fatty acid (NEFA), high-density lipoprotein cholesterol (HDL-C), low-density lipoprotein cholesterol (LDL-C), glucose, triglyceride (TG), creatinine, and blood urea nitrogen (BUN) were evaluated at a commercial laboratory (SRL, Tokyo).

### Gelatin Zymography

For gelatin zymography, 20 μg of aortic protein extract was mixed with sodium dodecyl sulfate (SDS) sample buffer without reducing agent and loaded onto a 10% SDS-polyacrylamide gel containing gelatin (1 mg/mL) containing 1 mg/mL gelatin as described as described in Lei et al. (2017). Following staining and destaining with the related buffers, the areas of gelatinolytic activity were visualized under a light microscope and the digestion bands were measured by an image analyzer software program (NIH Image 1.62).

### Western Blot Analysis

The total aortic protein was extracted with lysis buffer. The DC protein assay kit (Bio-Rad Laboratories, Hercules, CA, United States) was applied to measure the concentration of each sample. The protein abundance was detected with antibodies against Sirt-1 (cat. no. 2028, Cell Signaling Technology, Danvers, MA, United States), p16^INK4*A*^ (CDKN2A, cat. no. 10883-1-AP, Proteintech Group Inc., Rosemont, IL, United States), adiponectin receptor-1 (AdipR-1, ab70362), β-actin monoclonal antibody (1: 1000, AC-15, Sigma-Aldrich), and p21 (ab109199, both from Abcam, Cambridge, United Kingdom). The membranes then were incubated with the secondary antibodies. The protein contents calculated from western blots were normalized by loading β-actin.

### Preadipocyte Isolation and Culture

Inguinal adipose tissues of the non-stress and stressed mice-derived immature adipocytes were prepared as described previously (Hao et al., 2014). The preadipocytes were cultured in Dulbecco’s modified Eagle’s medium (DMEM)/10% fetal bovine serum (FBS), antibiotics (penicillin/streptomycin), and 4.5 g/l glucose in a humidified atmosphere (95% air and 5% CO_2__)_ at 37°C. Following culturing in serum-free medium for 12 h, the cells were applied to a biological assay.

### Statistical Analysis

The data are expressed as the mean ± standard error of the mean (SEM). We used Student’s *t*-test for comparisons of two groups, and we conducted a one-way analysis of variance (ANOVA) for comparisons of three or more groups followed by Tukey’s *post hoc* test. Probability (*p*)-values < 0.05 were considered significant. All of the examinations were performed by two observers blinded to the treatment of the animals.

## Results

### Effects of Chronic Stress on Body Weight, Plasma Lipid Profile, and Plasma GLP-1/APN Axis

**FIGURE 1:**
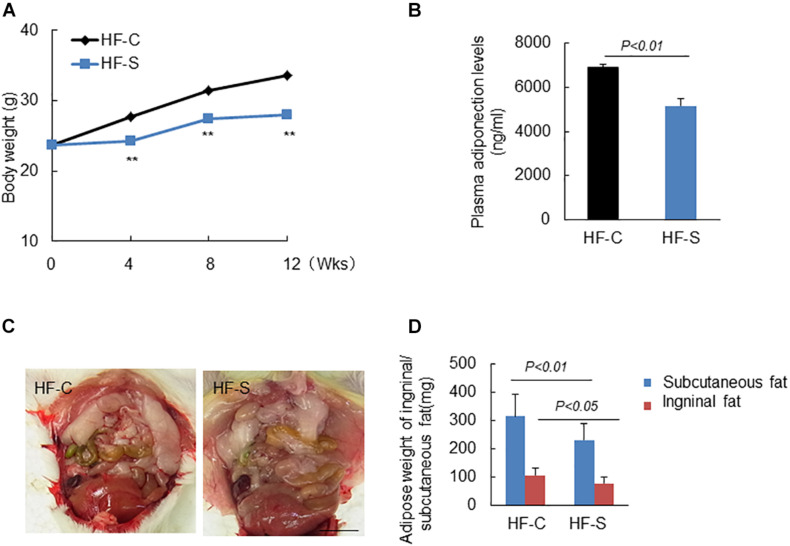
Body weight (BW), adipose weight and adiponectin levels in the two experimental groups during the experimental period. **(A)** Chronic stress reduced BW in a time dependent manner. **(B)** Chronic stress decreased serum APN levels. **(C,D)** Representative images show the inguinal fat, and chronic stress reduced the weight of subcutaneous and inguinal fat. Data are presented as means ± SEM (*n* = 6–8). *P* < 0.05 was considered statistically significant by Student’s un-paired *t*-test. Scale bars: 200 μm **(D)**.

The mice were weighed weekly and as expected, chronic stress significantly reduced the body weights of the stressed mice in a time-dependent manner compared to the non-stressed mice (Figure 1A) and also reduced the weight of subcutaneous and inguinal fat (Figures 1C,D). As shown in Table 2, except for TG levels, chronic stress had no effect on other plasma lipid profile levels or the blood glucose and BUN, CREA levels. However, the plasma ANP and GLP-1 levels of the stressed mice were significantly decreased as compared to the non-stress mice (APN: 6904.4 ± 124.3 ng/ml vs. 5167.1 ± 301.7 ng/ml; GLP-1: 15.2 ± 0.9pM vs. 11.7 ± 1.2pM, respectively; *p* < 0.01, Figure 1B and Table 2).

**TABLE 2:** Levels of lipids, GLP-1 activity, and other biochemical parameters.

**Parameter**	**HF-C**	**HF-S**
TG (mg/dL)	188.0 ± 4.7	113.5 ± 11.6*
LDL (mg/dL)	224.0 ± 18.4	228.5 ± 20.4
HDL (mg/dL)	28.0 ± 5.6	26.5 ± 4.8
NEFA (μEQ/L)	882.5 ± 26.3	793.5 ± 21.3
Glucose (mg/dL)	172.0 ± 16.4	161.5 ± 14.7
BUN (mg/dL)	18.0 ± 2.7	18.0 ± 3.1
CREA (mg/dL)	0.5 ± 0.0	0.5 ± 0.0
GLP-1 (pM)	15.2 ± 0.9	11.7 ± 1.2**

TG, triglyceride; LDL, high-density lipoprotein; HDL, high-density lipoprotein; BUN, blood urine nitrogen; CREA, creatinine; NEFA, non-esterified fatty acid; GLP-1, glucagon-like peptide-1. All results are presented as means ± SEM, *P < 0.05, **P < 0.01 vs. corresponding controls by Student’s un-paired *t*-test.

### Chronic Stress Accelerated the Diet-Induced Lipid Accumulation, Inflammatory Response and Oxidative Stress of Atherosclerotic Lesions

**FIGURE 2:**
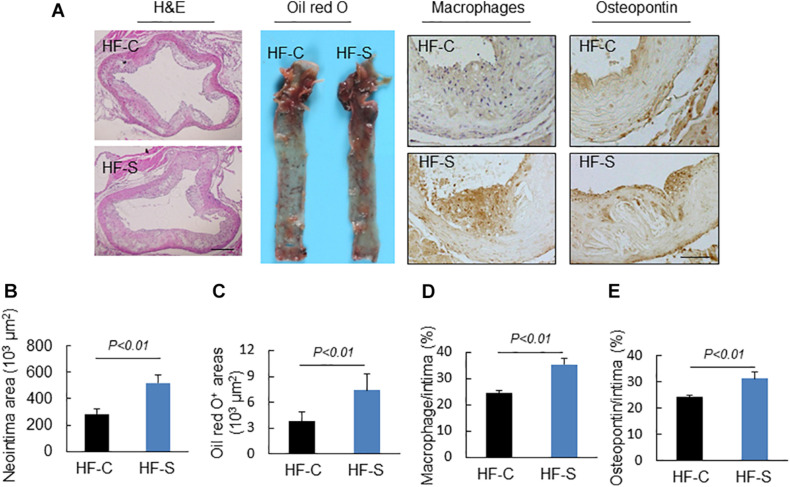
Histological characterization of atherogenic plaques in aortic roots of two experimental groups (high-fat diet alone, HF-C; high-fat diet plus stress, HF-S). **(A)** Representative images applied to evaluate the neointimal hyperplasia, content of lipid-rich plaques, macrophages accumulation and osteopontin expression. **(B–E)** The areas for H&E, Oil red O^+^ and positive areas in the neointimas for osteopontin and CD68 were quantified for each section. Results are presented as neointima area, the ratio of Oil red O-stained area to the total aortic root, the ratio of the positively stained area (CD68, osteopontin) to the neointimal area in the atherosclerotic lesions. Values are presented as means ± SEM (*n* = 6–7). *p* < 0.01 vs. HF-C by Student’s *t*-test. Scale bars: 50 μm.

As we expected, the chronic stress significantly increased the atherosclerotic lesion area in the aortic root compared to the control values (atherosclerotic area: 521.3 ± 57.6 × 10^3^ μm^2^ vs. 285.1 ± 36.2% × 10^3^ μm^2^, *p* < 0.01, Figures 2A,B), indicating that the chronic stress promoted atherosclerotic plaque expansion. Lipid deposition is known to be an initial process of atherosclerosis formation and development. Herein, we used oil red O staining to evaluate the lipid content of the plaques, which represents the severity of atherosclerosis. As shown in Figures 2A,C, the lipid accumulation in the atherosclerotic plaques was more severe in the stress groups compared to the controls. Clinically, after the lipid accumulation, the inflammation response and oxidative stress started to play critical roles in the development of atherosclerotic lesions. As shown in Figures 2A,D,E, chronic stress significantly increased the accumulation of macrophages (CD68: 35.5 ± 2.1% vs. 24.7 ± 0.8%, *p* < 0.01) and the expression of osteopontin proteins (osteopontin: 31.1 ± 0.8% vs. 24.2 ± 0.7%, *p* < 0.01) in the atherosclerotic plaques. The results of the RT-PCR confirmed that the expressions of the oxidative stress (p67^*phox*^, gp91^*phox*^, and p47^*phox*^), inflammation (MCP-1, TLR-2, TLR-4, and CXCR-4), and proteolysis (MMP-2, MMP-9, and Cat S)-related genes were markedly elevated in the aortas of the stressed mice compared to the control aortas (Table 3).

**TABLE 3:** Real-time PCR analysis of the targeted genes in the aortic roots and APN in subcutaneous fat of both experimental group mice.

**Parameter**	**HF-C**	**HF-S**
TLR-2	34.2 ± 0.9	41.5 ± 1.3**
TLR-4	37.8 ± 1.9	49.7 ± 2.1**
CXCR-4	71.6 ± 3.6	96.0 ± 3.3**
MCP-1	9.8 ± 0.5	15.2 ± 0.9**
gp 91^*phox*^	19.2 ± 0.8	26.3 ± 0.6**
p47^*phox*^	29.9 ± 1.5	35.8 ± 4.4*
P67^*phox*^	17.1 ± 0.5	22.7 ± 1.0**
Cat S	19.6 ± 1.5	29.3 ± 1.1**
MMP-2	26.7 ± 3.4	31.0 ± 3.5**
MMP-9	22.6 ± 3.1	46.0 ± 3.2**
APN	107.4 ± 22.8	69.2 ± 19.2**

TLR-2, toll-like receptor 2; CXCR-4, C-X-C chemokine receptor-4; MMP-2, matrix metalloproteinase-2; Cat S, cathepsin S; MCP-1, monocyte chemotaxis protein-1; APN, adiponectin. All results are presented as means ± SEM, *P < 0.05, **P < 0.01 vs. corresponding controls by Student’s un-paired *t*-test.

### Chronic Stress Prompted Vascular Senescence

**FIGURE 3:**
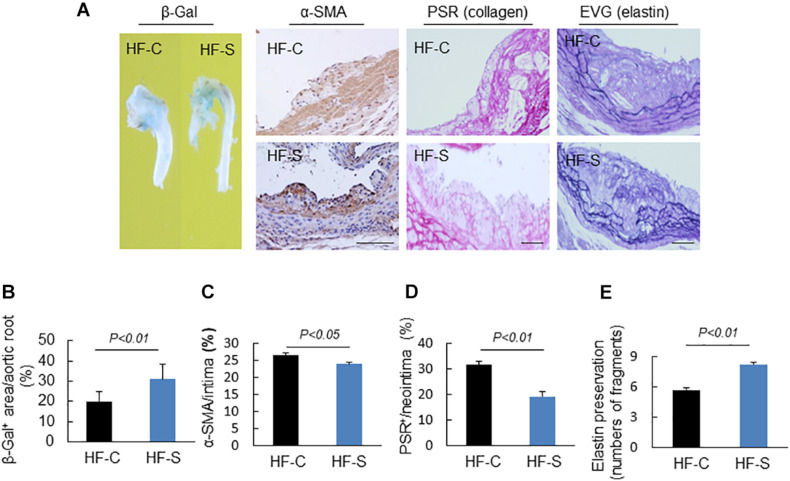
Morphological characterization of the plaques in aortic roots of two experimental groups. **(A)** Representative images applied to evaluate vascular senescence, α-SMCs, collagen, and elastin disruption. **(B–E)** The areas for β-Gal^+^, α-SMCs, PSR^+^ staining, and the elastic disruption degree in atherosclerotic lesions were calculated for each section. Results are presented as the ratio of β-Gal^+^ area to the total aortic root, the ratio of the positively stained area (α-SMCs, PSR^+^) to the neointimal area in the atherosclerotic lesions, and the elastic broken numbers in the atherosclerotic lesions. Values are presented as means ± SEM (*n* = 6–7). *p* < 0.01 vs. HF-C by Student’s *t*-test. Scale bars: 50 μm.

Endothelial dysfunction is a common pathological phenomenon for many cardiovascular diseases, and it often occurs before atherosclerosis. In this study, the senescence-associated β-gal staining showed that the positive staining area in the stressed mice was larger than that of the controls (Figures 3A,B), indicating that the chronic stress resulted in endothelial cell dysfunction.

### Chronic Stress Changed the Atherosclerotic Plaque Stability

Extracellular matrix (ECM) remodeling is responsible for the stability of atherosclerotic plaques, including the synthesis and degradation of collagen, elastin, and other glycoproteins. In this study, chronic stress significantly reduced the atherosclerotic plaques’ contents of collagen and α-SMC (the main source of collagen) compared to the control group (α-SMC: 26.6 ± 0.5% vs. 23.9 ± 0.6%, collagen: 31.6 ± 1.3% vs. 19.2 ± 1.9%, respectively; *p* < 0.05, *p* < 0.01; Figures 3A,C,D). The results of the EVG staining demonstrated that the chronic stress significantly destroyed the integrity of elastin in the media layer compared to the control group (number of breaks: 8.2 ± 0.3 vs. 5.7 ± 0.2, *p* < 0.01, Figures 3A,E).

### Pitavastatin Mitigated the Chronic Stress-Related Lipid Accumulation, Inflammatory Response, Oxidative Stress, and Plasma GLP-1/APN Axis

At the second phase of the experiment, we evaluated the effects of a statin, i.e., pitavastatin, on stress-related atherosclerosis. Pitavastatin significantly reduced the levels of LDL-C without changing the levels of the other parameters (TG, HDL, NEFA, glucose, BUN, CREA, and body weight) (Table 4). More importantly, we observed that pitavastatin significantly improved plasma adiponectin (APN: 7904.4 ± 664.1 ng/ml vs. 4980.3 ± 363.7 ng/ml; *p* < 0.01) and GLP-1 (GLP-1: 18.8 ± 1.2Pm vs. 13.9 ± 1.4pM, *p* < 0.01) levels of the stressed mice as compared to control mice (Table 4).

**TABLE 4:** Levels of lipids activity and other biochemical parameters.

**Parameter**	**Stress**	**Pitavastatin**
TG (mg/dL)	111.0 ± 10.4	108.5 ± 11.2
LDL (mg/dL)	213.5 ± 19.6	123.5 ± 13.2*
HDL (mg/dL)	24.5 ± 3.9	26.4 ± 3.7
NEFA (μEQ/L)	773.5 ± 20.8	712.2 ± 18.4
Glucose (mg/dL)	159.5 ± 13.9	158.6 ± 15.6
BUN (mg/dL)	19.0 ± 3.8	21.5 ± 5.1
CREA (mg/dL)	0.5 ± 0.0	0.5 ± 0.0
GLP-1 (pM)	13.9 ± 1.4	18.8 ± 1.2**
APN(ng/ml)	4980.3 ± 363.7	7904.4 ± 664.1**

Abbreviations are in Table 2. APN, adiponectin. Data are presented as means ± SEM, *P < 0.05, ** *P* < 0.01 vs. corresponding controls by Student’s un-paired *t*-test.

**FIGURE 4:**
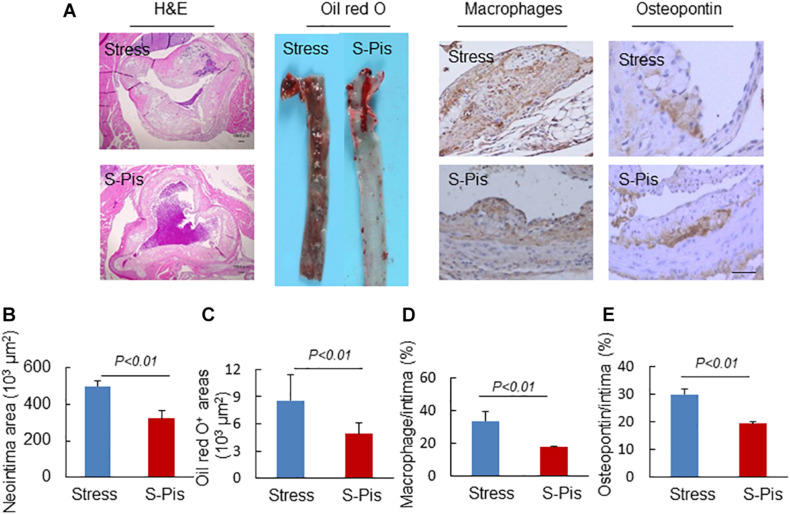
Morphological characterization of atherosclerotic plaques in aortic roots of the stress-alone and stress plus pitavastatin (S-Pis) groups. **(A)** Representative images used to assess the neointimal hyperplasia, content of lipid-rich plaques, macrophages accumulation and osteopontin expression. **(B–E)** The areas for H&E, Oil red O^+^, and positive areas in atherosclerotic neointimas for CD68 and osteopontin were calculated for each section. Results are expressed as neointima area, the ratio of Oil red O-stained area to the total aortic root, the ratio of the positively stained area (CD68, osteopontin) to the neointimal area in the atherosclerotic lesions. Values are presented as means ± SEM (*n* = 6–7). *p* < 0.01 vs. Stress group by Student’s *t*-test. Scale bars: 50 μm.

Pitavastatin treatment also reduced atherosclerotic area compared to the stress alone group (atherosclerotic area: 496.9 ± 28.1 × 10^3^μm^2^ vs. 321.2 ± 41.9 × 10^3^μm^2^; *p* < 0.01; Figures 4A,B). Compared to the non-treated stress group, the group of stressed mice treated with pitavastatin exhibited significantly reduced lipid deposition, macrophage accumulation, and osteopontin expression in the stress-related atherosclerotic lesions (Figures 4A,C–E). The mRNA expressions of p67^*phox*^, p47^*phox*^, gp91^*phox*^, CXCR4, MCP-1, TLR-4, and TLR-2 were inhibited (Table 5).

**TABLE 5:** Real-time PCR analysis of the targeted genes in the aortic roots and APN in subcutaneous fat of both experimental group mice.

**Parameter**	**Stress**	**Pitavastatin**
TLR-2	40.8 ± 2.6	22.3 ± 1.7**
TLR-4	46.7 ± 2.2	19.7 ± 0.9**
CXCR-4	87.0 ± 5.7	54.2 ± 4.0**
gp 91^*phox*^	27.1 ± 1.8	16.8 ± 0.9**
p47^*phox*^	32.7 ± 2.1	20.0 ± 1.1**
P67^*phox*^	23.6 ± 1.3	15.2 ± 0.8**
Cat S	26.9 ± 2.3	11.3 ± 1.1**
MMP-2	27.9 ± 3.6	24.1 ± 6.5*
MMP-9	39.8 ± 3.3	21.4 ± 0.8**
APN	76.4 ± 11.3	125.3 ± 27.4**

Abbreviations are in Table 3. Data are presented as means ± SEM, *P < 0.05, **P < 0.01 vs. corresponding controls by Student’s un-paired *t*-test.

### Pitavastatin Alleviated Vascular Aging and Atherosclerotic Plaque Instability

**FIGURE 5:**
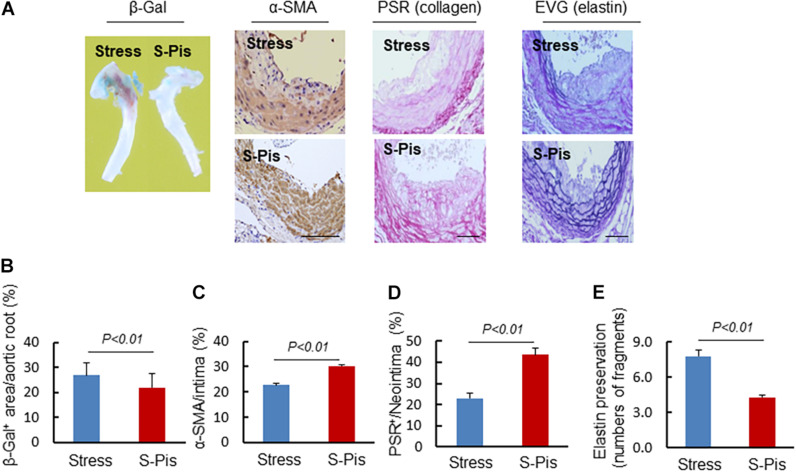
Histological characterization of atherosclerotic plaques in aortic roots of the Stress and S-Pis groups. **(A)** Representative images were used to assess vascular senescence, α-SMCs, collagen and elastin disruption, and. **(B–E)** The areas for β-Gal^+^, α-SMCs, PSR^+^ staining and the elastic disruption degree in atherosclerotic lesions were quantified for each section. Results are expressed as the ratio of β-Gal^+^ area to the total aortic root, the ratio of the positively stained area (α-SMCs, PSR^+^) to the neointimal area in the atherosclerotic lesions, and the elastic broken numbers in the atherosclerotic lesions. Values are presented as means ± SEM (*n* = 6–7). *p* < 0.01 vs. Stress group by Student’s *t*-test. Scale bars: 50 μm.

**FIGURE 6:**
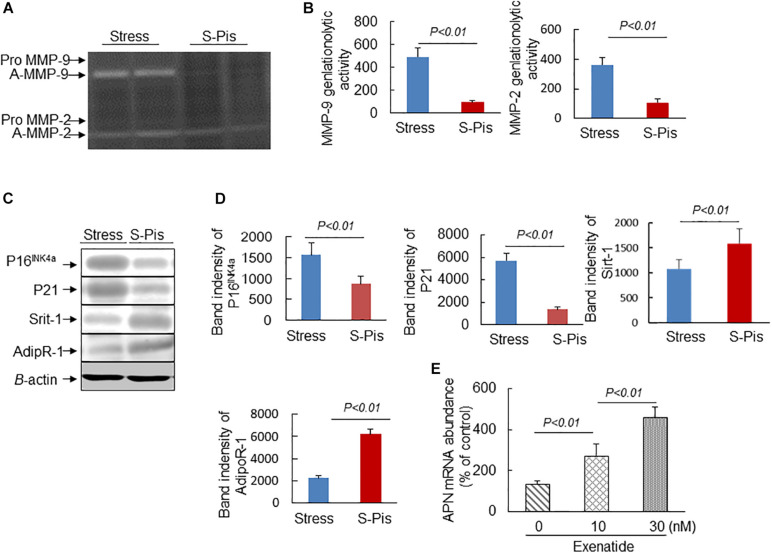
Pitavastatin mitigated MMP-2/-9 expression/activity and targeted protein expressions in the aortic roots. **(A,B)** Representative gelatin zymography images and quantitative data for MMP-9 and MMP-2 activities. **(C,D)** Representative Western blot images and quantitative data show the protein expressions of P16^*INK*4*a*^, P21, Sirt-1, and Adip R-1. **(E)** Exenatide elevated APN gene expression in the stressed mouse inguinal adipose tissue-derived immature adipocytes. Values are presented as means ± SEM (*n* = 3–6). *p* < 0.05, *p* < 0.01 vs. Stress group by Student’s *t*-test and one-way ANOVA followed by Tukey *post hoc* tests.

The vascular aging was clearly alleviated by the pitavastatin treatment, as shown in Figures 5A,B. In addition, the pitavastatin treatment increased the collagen and α-SMC contents of the atherosclerotic plaques and preserved the continuity of the elastic lamina (Figures 5A,C–E). To explore the cause of the destruction of the ECM, we determined the expressions and activities of ECM-degrading enzymes. The RT-PCR results revealed that the aortas of S-Pis mice had decreased levels of MMP-2, MMP-9, and Cat S mRNAs (Table 5). In addition, the gelatinolytic activity of MMP-9 and MMP-2 was also inhibited by pitavastatin treatment (Figures 6A,B).

It is known that APN is produced mainly by adipose tissue, and we therefore investigated the mRNA expression in the subcutaneous fat of the three groups of mice. As shown in Tables 3, 5, the chronic stress significantly inhibited the APN expression of the subcutaneous fat of the mice, and the pitavastatin treatment ameliorated the expression of APN. We also observed that the pitavastatin treatment mitigated the alterations in the senescence-related proteins (p16, p21, Sirt-1, and AdipR-1) (Figures 6C,D). We used an APN neutralizing antibody to test the anti-atherosclerotic effect of pitavastatin in the stressed mice, and the results showed that this antibody abolished the protective effects of pitavastatin in stress-related atherogenesis (Table 6). *In vitro*, exenatide as a GPL-1 receptor agonist increased APN gene expression in cultured immature adipocytes in a dose-dependent manner (Figure 6E).

**TABLE 6:** Histological characterization of atherosclerotic lesions in the aortic roots of both experimental group mice.

**Parameter (Aortic root, *n* = 6)**	**S-Pis-C**	**S-Pis-NAPN**
Intima (×10^3^/μm^2^)	308.4 ± 19.3	335.1 ± 4.2*
Media (×10^3^/μm^2^)	201.2 ± 26.0	219.3 ± 17.1
Intima/media ratio	1.6 ± 0.4	1.8 ± 0.6*

The ratio of intima to media was expressed as the ratio of intimal to media area in lesional cross-sections of heart aortic roots of HF-diet loaded stressed mice received pitavastatin (1mg/kg/d) plus rabbit IgG (Control, 450 μg/kg/d, S-Pis-C) or rabbit neutralizing APN antibody (450 μg/kg/d, S-Pis-NAPN). Data are presented as means ± SEM, *P < 0.05 was considered statistically significant.

## Discussion

In addition to conventional cardiovascular risk factors such as high cholesterol, hypertension, and diabetes mellitus, chronic stress is now considered a risk factor for cardiovascular diseases. Explorations of the underlying mechanisms and the identification of pharmacotherapeutic targets for clinical use are thus of great significance. Our present study’s findings may contribute to this field, as we observed the following: (a) Chronic stress significantly enhanced the inflammation action and oxidative stress process in *ApoE*^–/–^ mice fed a high-fat diet, and it increased endothelial senescence, which promoted the development of atherosclerosis. (b) Chronic stress favored the formation of vulnerable plaques by changing the components of the ECM, and it decreased the plaques’ α-SMC content. (c) As expected, the traditional anti-atherosclerosis drug pitavastatin alleviated the progression of atherosclerosis and promoted the stability of atherosclerotic plaques by ameliorating the vascular aging, inhibiting inflammation action and oxidative stress, and this effect may be exerted partly through the modulation of GLP-1/APN axis.

Atherosclerosis as an inflammatory disease and a great deal of evidence has confirmed the fundamental role of inflammation in the process of atherosclerosis (Zhu et al., 2018). The results of the present study demonstrated that chronic (12-week) stress significantly increased the accumulation of macrophages and the expression of osteopontin proteins of atherosclerotic plaques; the stress also increased the mRNA levels of TLR-2, TLR-4, MCP-1, and CXCR-4 in the mouse aorta. The inflammatory effects on atherosclerosis of these parameters have been fully proved by previous studies (Chiba et al., 2002; Cole et al., 2010; Cheng et al., 2011). Coincidentally, it was reported that statin treatment of *ApoE*^–/–^ mice inhibited an acute stress-related inflammation of atherosclerotic plaques (Janssen et al., 2015). In agreement with several previous studies (Isingrini et al., 2011; Hayashi et al., 2014; Heidt et al., 2014), the present data indicate that chronic stress accelerated the process of atherosclerosis by enhancing inflammatory action.

The negative effects of oxidative stress in atherosclerosis have been described, including endothelial cell dysfunction, foam cell formation, plaque disruption and their interaction with inflammation, all of which contribute to atherosclerosis (Linton et al., 2000; Lankin and Tikhaze, 2017). Our present findings revealed that chronic stress significantly increased the mRNA expressions of gp91^*phox*^, p47^*phox*^, and p67^*phox*^ in the aorta tissue. p67^*phox*^, p47^*phox*^, and gp91^*phox*^ are the components of NAD(P)H oxidases, which are the main source of reactive oxygen species (ROS) (Cai et al., 2003). It has been demonstrated that gp91^*phox*^ and p47^*phox*^ play critical roles in the development of atherosclerosis (Barry-Lane et al., 2001; Sorescu et al., 2002). In our present investigation, β-gal galactosidase staining revealed that chronic stress promoted the senescence of endothelial cells. Based on our present data, we speculate that chronic stress affects atherosclerosis in part by increasing the production of oxidative stress.

Plaque rupture is the leading cause of acute cardiovascular events, and such ruptures are always due to the formation of vulnerable plaques. An excessive inflammatory response, oxidative stress, and degradation of the ECM are all responsible for the formation of vulnerable plaques (Quesada et al., 2015; Ruddy et al., 2016; Morariu et al., 2019). As mentioned before, chronic stress promoted the progression of atherosclerosis in a mouse model by enhancing inflammation and oxidative stress, and the enhanced inflammation and oxidative stress can also change the characteristics of atherosclerotic plaques and promote ruptures. We also observed that chronic stress markedly decreased the atherosclerotic plaques’ collagen and α-SMC contents. The elastin integrity of the elastic laminae was destroyed under the condition of chronic stress, and it is known that the elastin, collagen and α-SMC contents are responsible for the stability of atherosclerotic plaques (Kumar et al., 2016; Yurdagul et al., 2016). Our results therefore indicate that the chronic stress changed the properties of the plaques, inducing the formation of vulnerable plaques.

Regarding the underlying mechanisms, we found that the chronic stress promoted the expressions of collagen-degrading enzymes (i.e., MMP-2, MMP-9, and Cat S) and also increased the activity of MMP-2 and MMP-9. The main function of members of the MMP (matrix metalloproteinase) family is to degrade and deposit structural proteins within the ECM, which would affect the plaque stability (Amin et al., 2016). Similarly to the MMP family, the cysteine proteases (which have collagenolytic and elastolytic activities) also involve in ECM degradation in the process of atherosclerosis—especially Cat K and Cat S (Sukhova et al., 2003; Lutgens et al., 2006). In the present study we noted that not only the expressions of MMPs and Cats but also the activities of MMP-2 and MMP-9 were significantly increased by chronic stress, suggesting that chronic stress changed the properties of atherosclerotic plaques via ECM-degrading enzymes.

The anti-atherosclerotic effects of statins have been well confirmed by basic research and in clinical practice. In addition to statins’ ability to reduce LDL-C levels, it has been demonstrated that statins exert other anti-atherosclerotic effects, including anti-inflammation, improving endothelial function, and reducing the production of ROS (Jarvisalo et al., 1999; Ascer et al., 2004; Ekstrand et al., 2015). The present study is the first to investigate the anti-inflammation and anti-oxidative stress effects of statins in stress-related atherosclerosis. Pitavastatin reduced the LDL-C levels in stressed *ApoE*^–/–^ mice and we also observed that: (a) Pitavastatin decreased the inflammatory response of the atherosclerosis by reducing the accumulation of macrophages and the expression of osteopontin proteins. The mRNA levels of CXCR4, MCP-1, and TLR-2/-4 of the atherosclerotic plaques were also decreased. (b) Pitavastatin decreased the mRNA expressions of gp91^*phox*^, p47^*phox*^, and p67^*phox*^, all of which are related to oxidative stress. (c) Pitavastatin enhanced the stability of atherosclerotic plaques by increasing the collagen and α-SMC contents in the plaques, and it reduced the expressions and/or activities of ECM-degrading enzymes (MMP-2, MMP-9, and Cat S), preserving the integrity of the elastic laminae. All of these effects of pitavastatin contributed to the alleviation of stress-related atherosclerosis, beyond lowering the LDL-C level. (d) Pitavastatin rectified the alterations in the senescence-related protein levels (p61, p21, and Sirt-1).

We studied the potential mechanisms underlying the effects of the chronic stress on atherosclerosis, and it is noteworthy that the pro-atherosclerosis effect of chronic stress was accompanied by a change in the level of GLP-1 and APN. Adipose tissue has been recognized as an important endocrine organ, capable of secreting various endocrine factors that modulate a wide variety of physiological functions (Fang and Judd, 2018). APN is one of the adipokines that has exhibited anti-inflammatory properties and an endothelial cell-protective effect. Several studies confirmed that APN can activate AMP kinase and NFκB activity in human aortic endothelial cells under hyperglycemic conditions, and intervention with APN resulted in a marked reduction of the atherogenic plaque area on the abdominal aorta in a murine model (Wu et al., 2017). Together with our research, the above-described findings indicate that chronic stress induced a significant decrease in the APN level, resulting in the aggravation of atherosclerosis. We also observed that the expression of AdipR-1 in the aortic root was reversed by the pitavastatin treatment and APN neutralizing antibody abolished the protective effects of pitavastatin in stress-related atherogenesis. Moreover, in immature adipocytes, GLP-1 receptor activation by the exenatide stimulated APN expression in a dose-dependent manner. We therefore speculate that the protective effect of pitavastatin in chronic stress-related atherosclerosis may depend in part on the GLP-1/APN pathway.

One major potential limitation of the present study is that although current studies contains *in vivo* APN depletion experiments and GLP-1-mediated regulation of APN expression in immature adipocytes, we could not conduct used genetic GLP-1 and APN animals to provide a direct evidence for the link between vascular aging and atherosclerosis and GLP-1/APN axis. Furthermore, it was proposed that chronic stress accelerated the oxidative stress of atherosclerotic lesions. Our findings for the oxidative stress were only based on the quantitative RT-PCR data analysis of the components of NAD(P)H oxidases. Additional data including the actual readout for oxidative stress in the vascular lesions the rescuing lesions in the presence of antioxidants, and an alteration in mitochondrial oxidative stress levels will be more convincing for exploring proposed mechanisms. Further study will be needed to investigate these issues.

## Conclusion

We observed that chronic stress aggravated high fat diet-induced atherosclerosis in a mouse model by inhibiting the GLP-1-mediated APN/adipoR1 pathway, and it subsequently enhanced the inflammation and oxidative stress process and changed the properties of atherosclerotic plaques. As anticipated, treatment with pitavastatin ameliorated this stress-related atherosclerosis. A greater understanding of the precise effects of chronic stress is of great importance for combating stress-related disorders. Although there are limitations to our present findings in mice, they demonstrate the influence of chronic stress on atherosclerosis, including potential mechanisms. Moreover, the beneficial effects of statins on chronic stress-related atherosclerosis were confirmed for the first time, which is meaningful in clinical treatment.

## Data Availability Statement

The original contributions presented in the study are included in the article/supplementary material, further inquiries can be directed to the corresponding author/s.

## Ethics Statement

The animal study was reviewed and approved by the Nagoya University Graduate School of Medicine.

## Author Contributions

YL: main contributor to the collection and assembly of data, manuscript drafting, biological and morphological analyses, data statistical analysis, and interpretation. LP, GY, AI, and XL: collection and assembly of samples and data. QC, HW, and MK: financial support and editing of the manuscript. XWC: main contributor to financial support, design, and editing of the manuscript. All authors approved the final version of submission.

## Conflict of Interest

The authors declare that the research was conducted in the absence of any commercial or financial relationships that could be construed as a potential conflict of interest.
